# Transcriptome analysis of duck embryo fibroblasts for the dynamic response to duck tembusu virus infection and dual regulation of apoptosis genes

**DOI:** 10.18632/aging.103759

**Published:** 2020-09-07

**Authors:** Yuhong Pan, Anchun Cheng, Xingcui Zhang, Mingshu Wang, Shun Chen, Dekang Zhu, Mafeng Liu, Xinxin Zhao, Qiao Yang, Ying Wu, Juan Huang, Shaqiu Zhang, Sai Mao, Xumin Ou, Qun Gao, Yanling Yu, Yunya Liu, Ling Zhang, Zhongqiong Yin, Bo Jing, Bin Tian, Leichang Pan, Mujeeb Ur Rehman, Xiaoyue Chen, Renyong Jia

**Affiliations:** 1 Research Center of Avian Disease, College of Veterinary Medicine, Sichuan Agricultural University, Chengdu 611130, Sichuan, China; 2 Institute of Preventive Veterinary Medicine, Sichuan Agricultural University, Chengdu 611130, Sichuan, China; 3 Key Laboratory of Animal Disease and Human Health of Sichuan Province, Chengdu 611130, Sichuan, China

**Keywords:** transcriptome, DTMUV, apoptosis, duck embryo fibroblast, dynamic response

## Abstract

Duck Tembusu virus (DTMUV) is an emerging pathogenic flavivirus that has caused enormous economic losses in Southeast Asia. However, the pathogenic mechanism and host’s responses after DTMUV infection remain poorly understood. During this study, total mRNA sequencing (RNA-Seq) analysis was used to detect the global gene expression in DEFs at various time points after DTMUV infection. We identified 326 genes altered significantly at all time points, and these genes were dynamically enriched in multifarious biological processes, including apoptosis, innate immune response, DNA replication, cell cycle arrest and DNA repair. Next, the results showed that apoptosis was induced and the proportion of apoptosis increased with time, and pro-apoptotic molecules caspases were activated. The RNA-seq data analysis further revealed that most pro-apoptosis and anti-apoptosis genes were early continually responsive, and the genes involved in both intrinsic and extrinsic apoptotic pathways were initiated. Further, the considerably enriched immune-relevant pathways were involved in apoptosis process, and protein-protein interactions (PPIs) analysis showed that IL6, STAT1, TNFAIP3, CFLAR and PTGS2 may be key regulators of DEFs apoptosis. In conclusion, this study not only contributes to understanding the underlying mechanism of DEFs infection with DTMUV, but also provides new insights into targets screening for antiviral therapy.

## INTRODUCTION

The Duck Tembusu virus (DTMUV) is an arbovirus belonging to the genus *Flavivirus*, family *Flaviviridae*. DTMUV is a single-stranded, positive-polarity RNA flavivirus with a ~11 kb genome, which has only one open reading frame (ORF). The ORF encodes a polyprotein of ~3400aa (amino acid) residues, which is subsequently cleaved into three structural proteins (capsid [C]; precursor of M [prM] and envelope [E]) and seven nonstructural (NS) proteins (NS1, NS2A/2B, NS3, NS4A/4B and NS5) [1–6].

In recent years, DTMUV has caused huge economic losses to the poultry industry in Southeast Asian countries such as Thailand, Malaysia and China [7, 8]. In addition to infecting ducks, DTMUV can also infect chickens, geese, pigeons and house sparrows [9–11]. *In vivo* experiments showed that DTMUV can replicate in the spleen, kidneys and brains of BALB/c mice and Kunming mice, and cause systemic infection after intracerebral inoculation [12, 13]. A wide spectrum of mammalian cells is susceptible to DTMUV and exhibit cytopathic effects (CPEs), such as Vero, BHK21 and Hela [14]. Moreover, more than 70% of duck industry workers were reported to have Abs against DTMUV in the serum samples tested, and ~50% of oral swab samples were found to be positive for DTMUV RNA [15]. It is worth noting that flaviviruses such as BAGV [16] and TMUV [17, 18], which cross-react with DTMUV, can infect human beings. These studies clearly prove that DTMUV is likely to spread from ducks to other non-avian hosts and even humans [15]. Hence, DTMUV is very likely to become a zoonotic pathogen, and it is urgent to carry out antiviral research on DTMUV.

Apoptosis, also known as programmed cell death (PCD), is a crucial process for the host to resist pathogens invasion [19]. Apoptosis classically occurs via the intrinsic and extrinsic apoptotic pathways [20]. The intrinsic apoptosis pathway includes mitochondria- and endoplasmic reticulum (ER)-activated apoptosis. The mitochondrial apoptotic pathway could be initiated by numerous factors, such as nutrient deprivation, hypoxia and oxidative stress, resulting in a decrease in mitochondrial membrane potential (MMP) [21]. Subsequently, cytochrome C (cyt-c) is released to cytoplasm, a process closely controlled by the Bcl-2 protein family. Bcl-2 family proteins are divided into pro-apoptotic proteins (Bak, Bad, Bax, Bid) and anti-apoptotic proteins (Bcl-xl, Bcl-2, Mcl-1) [22]. Cyt-c can recruit pro-caspase-9 and apoptotic protease activating factor-1 (Apaf-1) to form an apoptosome, which then activates downstream caspase-3/7 to trigger apoptosis. The extrinsic apoptosis pathway, also called the death receptor pathway, is initiated by the binding of the death ligand to the corresponding receptor. Next, pro-caspase-8 is recruited to form a death-inducing signaling complex (DISC), resulting in the activation of caspase-8 and caspase-3/7, which ultimately leads to cleavage of cellular DNA [23, 24]. Although apoptosis can inhibit viral replication, many viruses have evolved strategies to prevent the occurrence of apoptosis during viral replication until sufficient progeny viruses are produced to enhance the spread of the virus [25, 26]. Therefore, understanding the mechanism of DTMUV regulating apoptosis is of great significance for future research.

Emerging technologies such as transcriptomics have become crucial tools for studying the pathogenesis of virus-infected host cells [27–29]. Transcriptome analysis can detect all RNA transcripts in cells, helping to clarify the expression levels of genes in different cellular environments [30]. Moreover, several important viruses have applied this technique to study the molecular mechanisms of pathogen-host interactions, including hepatitis E virus (HEV) [31], dengue virus (DENV) [10, 32], influenza A virus [33], Zika virus (ZIKV) [34] and Zaire Ebola virus (ZEBOV) [35]. These studies not only help to understand the host response after viral infection, but also provide clues for exploring the potential targets of antiviral drugs. Nevertheless, until now, information about the responses of host cell DEFs to DTMUV infection is rarely addressed.

In this study, in order to study the interaction between DTMUV and host cells, we used transcriptome approach to compare the genome-wide expression of the DTMUV-infected groups and the mock-infected groups at 12, 24, 36, 48 and 60 hpi (hours post infection). Using the Gene Ontology (GO) and Kyoto Encyclopedia of Genes and Genomes (KEGG) enrichment analyses, differentially expressed genes (DEGs) at different stages of infection were screened out. Eventually, we focused on the apoptotic pathway and the dynamic changes of pro-apoptosis and anti-apoptosis genes in DTMUV-infected DEFs. In conclusion, these findings provide prime information for a deeper understanding of the host’s response to DTMUV infection and the development of strategies to control DTMUV infection.

## RESULTS

### Characteristics of DEFs infected with DTMUV

**Figure 1:**
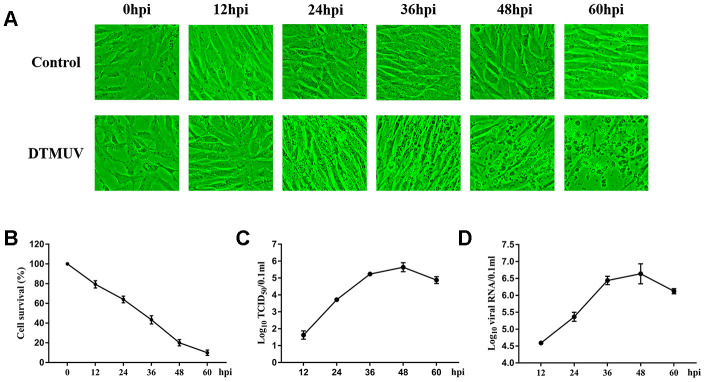
**DTMUV infection in DEFs cells.** (**A**) The cytopathic effects (CPE) of DEFs cells at 12, 24, 36, 48 and 60 hpi, and mock-infected cells as control. (**B**) The cell survival analysis at 12, 24, 36, 48 and 60 hpi. (**C**) One-step growth curve of DTMUV in DEFs cells. (**D**) Quantitative analysis of viral DNA by quantitative real-time PCR assay.

To determine the proliferation kinetics of DTMUV in DEFs, CPEs and the viral titers were detected at different time points after infection. In Figure 1A, minimal CPEs can be observed at 24 hpi and obvious CPEs appeared at 36 hpi, such as increased granularity and cellular fragmentation, and almost all cells fell off at 60 hpi. In Figure 1B, the viability of DEFs gradually decreased with the extension of viral infection time, confirming the high efficiency of viral infection. We used median tissue culture infective dose (TCID_50_) assays and Q-RT-PCR to monitor DTMUV proliferation (Figure 1C, 1D); the results showed that as the infection progressed, both the titers and viral RNA gradually increased.

### Global changes of gene expression after DTMUV infection

**Figure 2:**
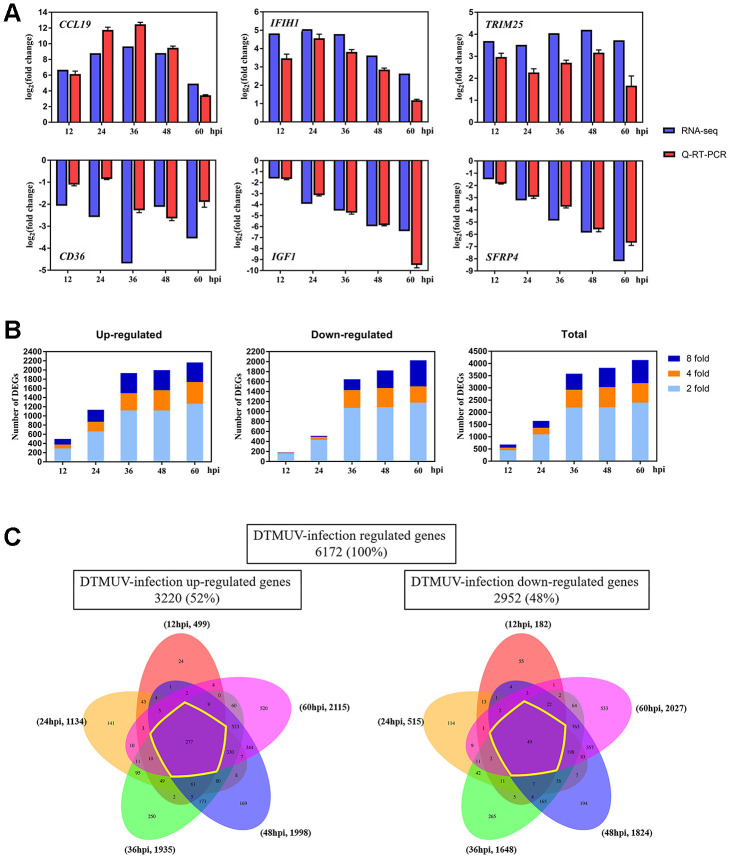
**Changes of the gene expression in DEFs at different times after DTMUV infection.** (**A**) Q-RT-PCR versus RNA-seq analyses of the expression for representative six genes (*CCL19*, *IFIH1*, *TRIM25*, *CD36*, *IGF1* and *SFRP4*). (**B**) The upregulated/downregulated number and total number of DEGs (≥ twofold change, *P*<0.05) at 12, 24, 36, 48 and 60 hpi. (**C**) Venn diagrams showing overlap of DTMUV-induced DEGs across different time points. Upregulated and downregulated genes were analyzed separately and have been shown with the number of genes specifically or commonly responsive at different time points.

As shown in Supplementary Dataset 1, genes with 2-fold changes or greater at 12, 24, 36, 48 and 60 hpi were defined as DEGs (*P*<0.05). In Figure 2A, six randomly selected DEGs were verified by Q-RT-PCR and confirmed that they all have good similarity to RNA-seq results, indicating that our data is accurate and valid and hence can be used for biological analysis. The principal component analysis (PCA) can demonstrate the dissimilarities among different samples. In Supplementary Figure 1, all the mock-infected samples were clearly distinct from DTMUV-infected samples, and the DTMUV-infected samples at each time point did not cluster with other time point. All sequencing data were deposited online in SRA (accession number SRS6277135).

Next, we compared the transcriptome profiles of DTMUV-infected groups relative to mock-infected groups at different time points to determine the DEGs levels during DTMUV infection. In Figure 2B, the numbers of DEGs increased with the time of DTMUV infection (681, 1649, 3583, 3822 and 4142 at 12, 24, 36, 48 and 60 hpi, severally), and most DEGs were upregulated or downregulated after 24 hpi. Since there were numerous DEGs at each time point, we first screened 10 genes that were most upregulated or downregulated (log_2_foldchange) at various time points for analysis (Table 1).

**Table 1:** The genes that were most up- or downregulated at each time point of DTMUV infection.

**Gene category**	**12hpi**		**24hpi**		**36hpi**		**48hpi**		**60hpi**	
	**Gene**	**log_2_FoldChange**	**Gene**	**log_2_FoldChange**	**Gene**	**log_2_FoldChange**	**Gene**	**log_2_FoldChange**	**Gene**	**log_2_FoldChange**
Upregulated	*RSAD2*	9.66	*RSAD2*	10.31	*POMC*	12.64	*IL12B*	12.22	*HMX3*	10.72
	*DDX60*	8.96	*CMPK2*	9.55	*RSAD2*	11.78	*IFITM1*	10.71	*POMC*	9.91
	*EPSTI1*	8.46	*DDX60*	9.39	*IL12B*	11.72	*POMC*	10.59	*RSAD2*	9.88
	*CMPK2*	8.13	*IL12B*	9.17	*LHX4*	11.21	*RSAD2*	10.55	*CMPK2*	9.14
	*USP18*	7.86	*CCL19*	8.79	*CMPK2*	11.2	*HMX3*	10.37	*SIX3*	9.04
	*CD7*	7.09	*LHX4*	8.58	*SIX3*	11.14	*EOMES*	10.06	*IFITM1*	9.02
	*VCAM1*	6.7	*IFITM1*	8.55	*HMX3*	10.68	*CMPK2*	10.06	*THEMIS*	8.29
	*CCL19*	6.67	*EPSTI1*	8.54	*FLT3*	10.68	*LHX4*	10.06	*FGF4*	8.28
	*IFITM1*	6.65	*USP18*	8.39	*CD7*	10.54	*NKX2-1*	9.42	*DDX60*	8.26
	*TRANK1*	6.45	*POMC*	8.03	*THEMIS*	10.18	*FOXS1*	9.2	*USP18*	8.21
										
Downregulated	*HPD*	-3.76	*SIGLEC15*	-4.65	*LMOD3*	-7.55	*MLXIPL*	-8.19	*C7H21orf58*	-10.06
	*ACSL5*	-3.34	*CER1*	-4.26	*GJD4*	-6.34	*MARCO*	-7.26	*ADPRHL1*	-9.61
	*ADRA1B*	-3.27	*CD36*	-3.93	*ANKRD34B*	-5.96	*GJD4*	-7.12	*ENPEP*	-8.66
	*CD79B*	-3.13	*TLDC2*	-3.84	*C1QTNF8*	-5.92	*ADPRHL1*	-7.05	*MARCO*	-8.39
	*HHATL*	-2.61	*SLC5A12*	-3.59	*IGSF10*	-5.6	*ENPEP*	-7.01	*C7*	-8.24
	*CD4*	-2.59	*PAH*	-3.52	*OMG*	-5.54	*P2RY13*	-6.87	*IGF1*	-8.2
	*NR4A1*	-2.56	*WEE2*	-3.33	*TLR4*	-5.17	*CACNG1*	-6.54	*MLKL*	-8.08
	*FMOD*	-2.38	*GDF2*	-3.28	*CMBL*	-5.08	*PPP1R3A*	-6.48	*MGP*	-8.06
	*MEF2B*	-2.34	*IGF1*	-3.22	*C4H4orf54*	-5.05	*C7H21orf58*	-6.47	*HSPB2*	-7.54
	*TNXB*	-2.33	*IGSF10*	-3.14	*MARCO*	-4.97	*C4H4orf54*	-6.4	*CACNG1*	-7.53

Among the upregulated genes, *RSAD2* (radical S-adenosyl methionine domain-containing protein 2) was most significantly upregulated at 12 and 24 hpi, while *POMC* (proopiomelanocortin), *IL12B* (interleukin 12B) and *HMX3* (H6 family homeobox 3) were most upregulated at 36, 48 and 60 hpi, respectively (Table 1). It’s worth noting that the interferon-stimulated gene *RSAD2* was significantly upregulated at all the time points, indicating the activation of antiviral and innate immune responses.

Among the downregulated genes, *HPD* (4-hydroxyphenylpyruvate dioxygenase), *SIGLEC15* (sialic acid binding Ig like lectin 15), *LMOD3* (leiomodin 3), *MLXIPL* (MLX interacting protein like) and *C7H21orf58* (chromosome 7 C21orf58 homolog) were most downregulated at 12, 24, 36, 48 and 60 hpi, respectively (Table 1).

Further, we established Venn diagrams to delve deeper into genes that are unique or shared at each time point (Figure 2C). In total, 6172 DEGs were identified after DTMUV infection, of which 3220 genes were upregulated (52%) and 2952 genes were downregulated (48%). Of the 3220 upregulated genes, 499 were upregulated at 12 hpi, 1134 at 24 hpi, 1935 at 36 hpi, 1998 at 48 hpi and 2115 at 60 hpi; among 2952 downregulated genes, 182 were downregulated at 12 hpi, 515 at 24 hpi, 1648 at 36 hpi, 1824 at 48 hpi and 2027 at 60 hpi. In the meantime, most differentially expressed genes were activated only at the 60 hpi, 520 out of 3220 upregulated genes (16.15%) compared with 24 (0.75%), 141 (4.38%), 250 (7.76%) and 169 (5.25%) at 12, 24, 36 and 48 hpi, similarity, 533 out of 2952 downregulated genes (18.06%) compared with only 55 (1.86%), 114 (3.86%), 265 (8.98%) and 194 (6.57%) at 12, 24, 36 and 48 hpi, respectively.

### GO and KEGG enrichment analysis

**Figure 3:**
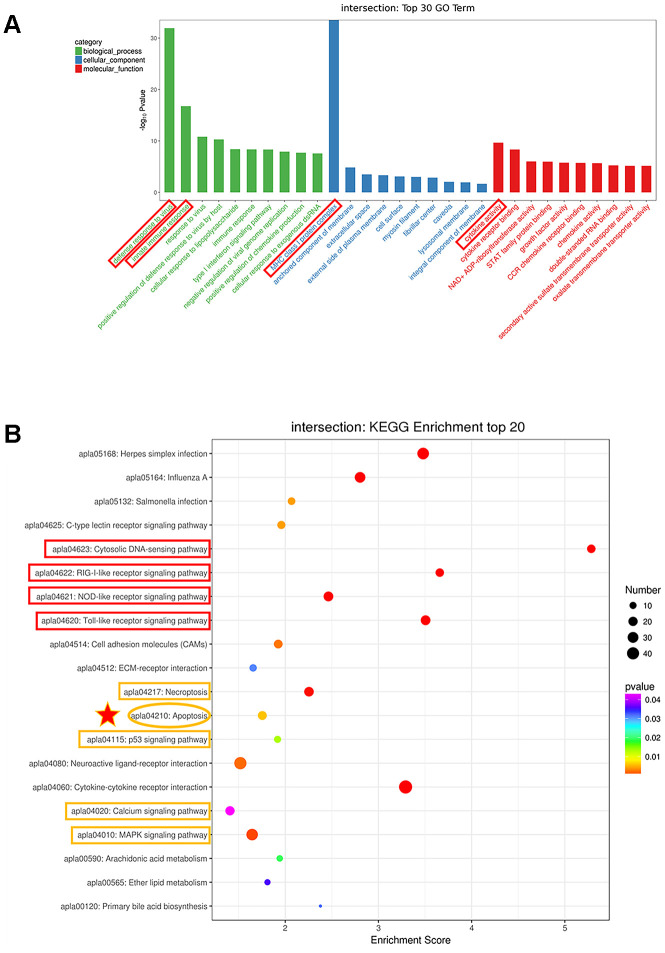
**Analysis of the genes with expression changes at all time points.** (**A**) The top 30 Gene Ontology (GO) enrichment of differentially expressed genes. (**B**) The top 20 Kyoto Encyclopedia of Genes and Genomes (KEGG) enrichment analysis of differentially expressed genes.

In Figure 2C, genes that were differentially expressed at all time points were called continuous upregulated and downregulated genes, marked with yellow circle, the DEGs in this group undoubtedly play an important role during DTMUV infection. The Venn diagrams revealed that 277 genes were continuous upregulated, whereas only 49 genes were continuous downregulated. In Figure 3A, a total of 326 genes that were differentially expressed at all time points were subjected to GO enrichment analysis, and it was found that the DEGs were mainly related to defense response to virus, innate immune response, MHC class I protein complex and cytokine activity. In addition, the details of GO enrichment analysis are shown in Supplementary Dataset 2. In addition, the results of GO analysis of DEGs that were upregulated or downregulated at all time points are shown in Supplementary Figures 2A, 3A, respectively.

What’s more, to delve into the functions of these DEGs, KEGG enrichment analysis was performed. In Figure 3B, DEGs were considerably enriched in immune-relevant pathways, including the Toll-like receptor signaling pathway, NOD-like receptor signaling pathway, RIG-I-like receptor signaling pathway and cytosolic DNA-sensing pathway, which plays a vital role in controlling viral infection and antiviral immune regulation. In addition, a strong enrichment was observed for genes involved in apoptosis, indicating that dysregulation of cell survival and cell growth are associated with DTMUV infection. The DEGs were also enriched in p53 signaling pathway that positively regulates apoptosis and cell cycle arrest, suggesting that DTMUV will induce cell growth arrest. Moreover, Necroptosis, Calcium signaling pathway and MAPK signaling pathway, which are involved in Apoptosis, were also enriched. In addition, the details of KEGG enrichment analysis are shown in Supplementary Dataset 3. Similarly, the results of KEGG analysis of DEGs that were upregulated or downregulated at all time points are shown in Supplementary Figures 2B, 3B, respectively.

### Differential expression of innate immune cytokines in DTMUV-infected DEFs

**Figure 4:**
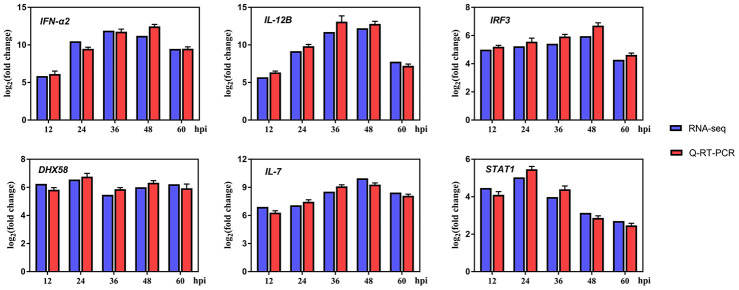
**Differentially expressed genes (DEGs) in innate immune system-related response after infection.**

A variety of evidences indicate that innate immune responses plays an important role in limiting flavivirus infection [42, 43]. In the GO and KEGG enrichment analyses, the DEGs were significantly enriched in immune-related pathways (Figure 3A and 3B), moreover, in Table 1, we found that the interferon-stimulated gene *RSAD2* was significantly upregulated, so it was of interest to survey the transcriptomic information about key genes in immune-related pathways. Thus, we examined the expression levels of cytokines participated in immune-related pathways, specifically, *IFN-α2*, *IL12B*, IFN regulatory factor 3 (*IRF3*), *DHX58*, *IL-7*, and signal transducer and activator of transcription 1 (*STAT1*) were selected. As shown in Figure 4, the results of both Q-RT-PCR and RNA-seq showed that all these genes are upregulated to varying degrees at various time points. Hence, these results indicated that innate immune responses were robust after DTMUV infection, which is consist with the GO and KEGG enrichment results. Since the elimination of virus-infected cells by apoptosis is often affected by the innate immune responses, the innate immune responses may be involved in DTMUV-induced apoptosis.

### DTMUV induces the counteraction of pro-apoptosis and anti-apoptosis

**Figure 5:**
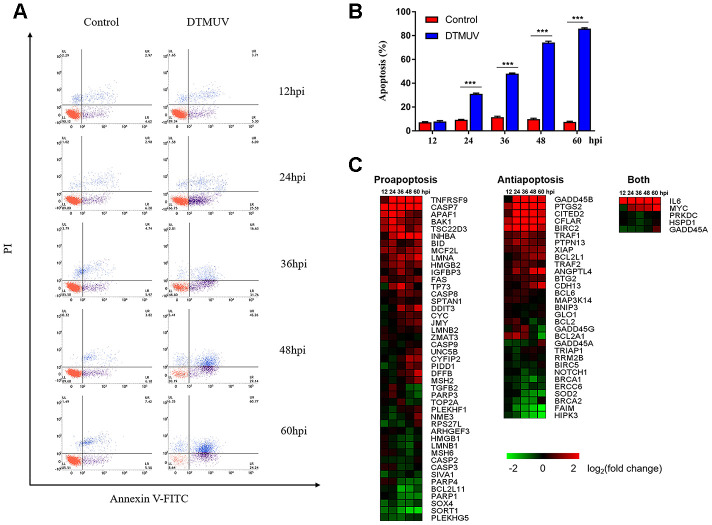
**Apoptotic effects induced by DTMUV infection.** (**A**) The absence of apoptosis analyzed by double staining with annexin V-FITC/PI, followed by flow cytometry. Representative images are shown (*n*=3). (**B**) Histogram of the percentage of apoptotic cells. The data are presented as the means ± SD of three independent experiments. *** *p*<0.0001, compared with the control group. (**C**) The heat map illustrating the dynamic regulation of apoptosis genes by DTMUV infection, including genes involved in pro-apoptosis, anti-apoptosis, or both positive and negative regulation of apoptosis. The green-to-red gradient bar represents log_2_ values of fold-changes in the gene expression induced by DTMUV infection.

Apoptosis play a vital role in virus-induced cytotoxicity [44]. Analysis of the sequencing data indicated that apoptosis pathway was activated after DTMUV infection. In order to link the analysis results with virus biology, we infected DEFs with DTMUV, and the flow cytometric analysis was performed after staining with Annexin V-FITC and PI, the results showed that DTMUV had a significant pro-apoptotic effect and obvious time dependence (Figure 5A, 5B).

Based on GO analysis, we identified 81 genes related to apoptosis, of which 45 and 31 genes play a positive and negative regulation role, respectively, and another 5 genes may regulate apoptosis either in both manners or in an unclear way (Supplementary Dataset 4). Meanwhile, the changes in gene expression shown by the heat map indicated that DTMUV infection will alter both pro-apoptosis and anti-apoptosis genes (Figure 5C). Among pro-apoptosis genes, death receptor genes (TNFRSF9 and FAS), mitochondrial pathway related genes (caspase-9, cyt-c and apaf-1), Bcl-2 family pro-apoptosis genes (BAK1 and BID, inhibitors of the IAP family members) and p53-dependent target genes (CYFIP2), were upregulated at various time points after DTMUV infection. The results indicate that both intrinsic and extrinsic apoptosis pathways may be initiated. Furthermore, anti-apoptotic genes, GADD45B and CFLAR, and the IAP family members (BIRC2 and BCL2L1) were upregulated early or late after DTMUV infection. Although the expression of the pro-apoptotic and anti-apoptotic genes were both altered, genes involved in pro-apoptotic in the competition between the two ultimately had an advantage, thus DTMUV infection induced apoptosis.

**Figure 6:**
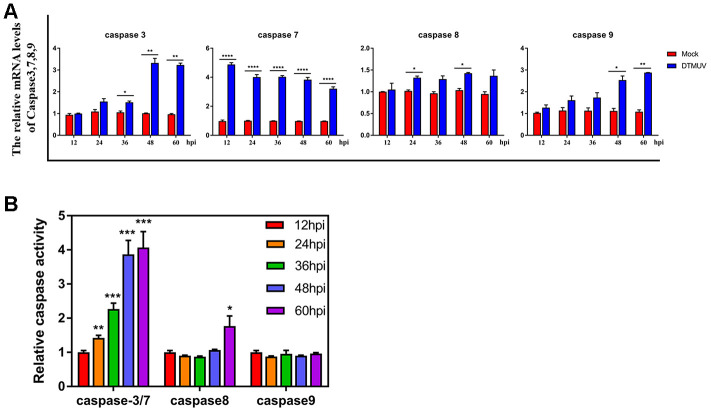
**Effects of DTMUV infection on the caspase family.** (**A**) mRNA expression levels of caspase-3, caspase-7, caspase-8 and caspase-9. (**B**) Activities of caspase-3, caspase-7, caspase-8 and caspase-9. The data are presented as the means ± SD of three independent experiments. * *p*<0.05, ** *p*<0.01 and *** *p*<0.0001, compared with the control group.

In addition, to determine whether the caspases family protein plays an indispensable role in DTMUV-induced apoptosis, we detected the mRNA levels of caspase-3/7/8/9 by Q-RT-PCR. In Figure 6A, caspase-7 mRNA levels in DTMUV-infected cells increased significantly at all time points, while the caspase-3 mRNA levels were significantly increased at 36, 48 and 60 hpi. What’s more, caspase-8 mRNA levels increased observably at 24 and 48 hpi, while caspase-9 mRNA increased significantly at 48 and 60 hpi. Figure 6B showed that the enzymatic activity of caspase-3/7 in the infection groups increased significantly at all the time points compared to the control groups, while the caspase-8 enzymatic activity only increased significantly at 60 hpi. In contrast, the caspase-9 enzymatic activity did not change significantly at all the time points. These findings suggested that caspases were involved in the apoptosis triggered by DTMUV in DEFs.

### DTMUV induces DEF S-phase cell cycle arrest

**Figure 7:**
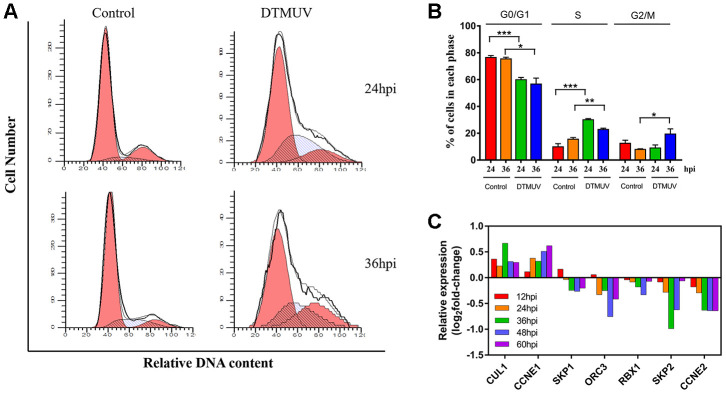
**Analysis of cell cycle arrest induced by DTMUV infection.** (**A**) The representative distribution and (**B**) the percentage of cells in G0/G1, S and G2/M phases at 24 and 36 hpi. Histograms of the cell percentage data are also shown (n=3). * *p*<0.05, ** *p*<0.01 and *** *p*<0.0001, compared with the control group. (**C**) RNA-seq data showing transcriptional expression changes of some key factors in the S checkpoint at 12, 24, 36, 48 and 60 hpi.

Studies have shown that DNA damage caused by viral infections can lead to cell cycle arrest [45]. Analysis of the results of flow cytometry showed that the cells number of S phase at 24 and 36 hpi in the DTMUV-infected group was significantly higher than that in the control group (Figure 7A, 7B), indicating that the cell blockage in S phase after DTMUV infection. In addition, we found that DTMUV can induce downregulation of numerous genes involved in DNA replication, thereby inhibiting cell cycle progression (Supplementary Table 1). Further, the key genes of S phase were tested. CUL1 and CCNE1, two S phase target genes, whose mRNA levels were upregulated at various time points after DTMUV infection, while the mRNA levels of RBX1, SKP2 and CCNE2 were decreased at all the time points, moreover, CCNE2 was time-dependently downregulated. Further, the expression levels of SKP1 and ORC3 were increased only at 12 hpi, but decreased at 24, 36, 48 and 60 hpi (Figure 7C). Interestingly, G2/M phase arrest only appeared at 36 hpi, but not at 24 hpi (Figure 7B). As we can see, the proportion of S phase arrested cells at 36 hpi was significantly lower than that at 24 hpi, possibly because with the prolonged infection time, some S phase cells entered G2/M phase, thus the cells blocked in G2/M phase at 36 hpi was significantly increased. Since the G2/M phase can protect cell viability by giving time for DNA repair [46], we analyzed the expression of genes participated in DNA repair pathways (Supplementary Table 2), containing mismatch repair, base excision repair, recombinational repair and nucleotide excision repair. It was found that most genes were significantly suppressed, indicating that the inefficiency of DNA repair after DTMUV infection.

### PPI revealed key genes regulating DEFs apoptosis

**Figure 8:**
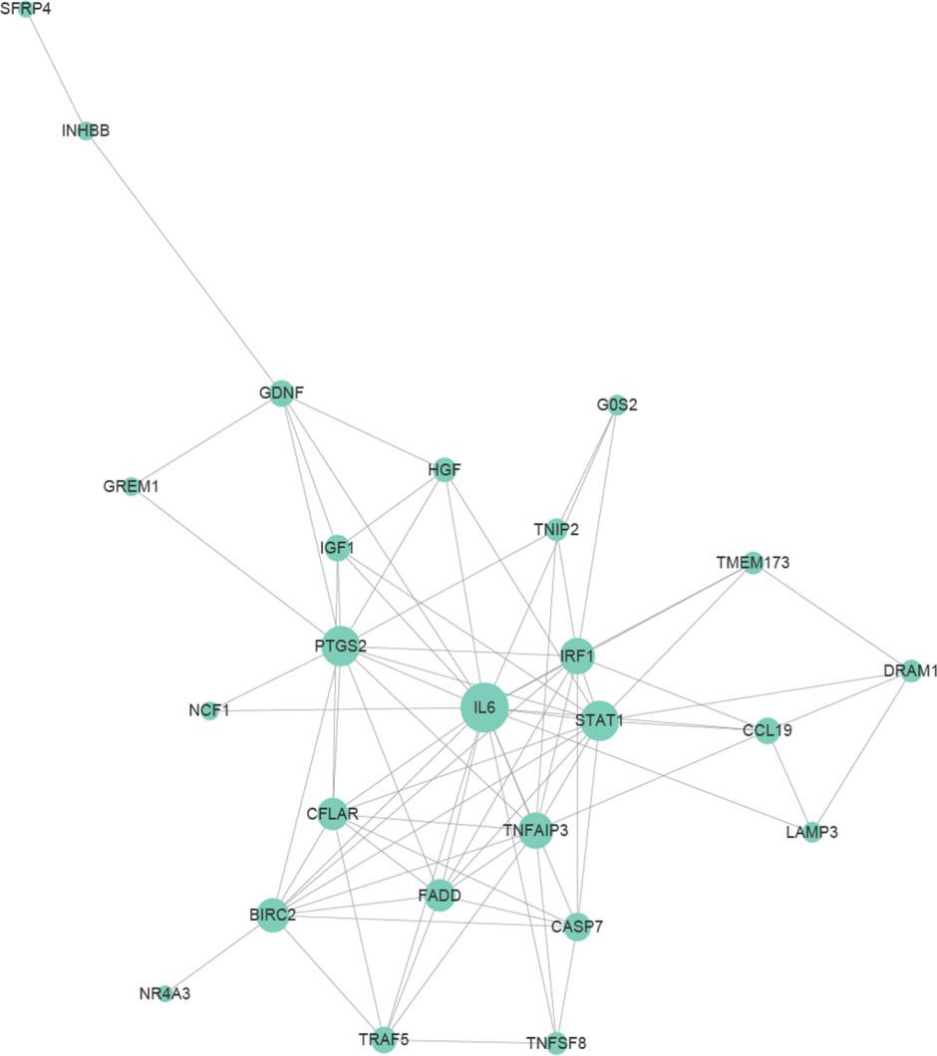
**Protein to protein interaction (PPI) relationships of apoptosis-related genes.** Apoptosis-related genes were selected from genes differentially expressed at various time points that were annotated with “apoptosis” and had significant.

In order to explore key genes involved in the regulation of DEFs apoptosis, we performed PPIs (protein to protein interactions) analysis using genes related to apoptosis that were significantly differentially expressed at all the time points (*P*<0.05). This PPI network diagram was used to reveal the central genes involved in the apoptosis of DEFs (Figure 8). Genes with higher edge count, such as IL-6 [47], STAT1 [48], TNFAIP3 [49], CFLAR [50], IRF1 [51] and PTGS2 [52], may be the central nodes of the apoptotic network.

## DISCUSSION

DTMUV is a vital pathogen that harms waterfowl and has caused huge economic losses to the poultry industry in China since 2010. Hence, deepening the understanding of the molecular mechanism of host-pathogen interactions is of great significance to inhibit the occurrence and prevalence of DTMUV infection. In recent years, RNA-Seq technology has become a powerful and revolutionary tool for uncovering molecular expression profiles [53–55]. Nevertheless, little information is available on the molecular expression profiles of DTMUV interacting with host cell DEFs.

In this research, to better elucidate the precise molecular mechanisms of DTMUV invasion into host cells, DEFs were served as an *in vitro* model to analyze the global molecular expression profiles. In total, 6172 DEGs were identified, of which 326 genes were continuous upregulated or downregulated at all time points (Figure 2C). Specifically, *RSAD2* was most upregulated at the intersections of all time points (Table 1). *RSAD2* is an interferon-stimulated gene involved in innate immunity and subsequent adaptive immunity during viral infection, as such is mainly responsible for limiting viral replication and antiviral responses [56–58]. In addition, *RSAD2* had been shown to limit the replication of multiple viruses in monocytes, fibroblasts and neurons, such as DENV [59], HCV [60], WNV [61], chikungunya [62], influenza [63], human cytomegalovirus [56] and human immunodeficiency viruses [64]. Thus, the upregulation of *RSAD2* is likely to prevent DTMUV invasion by activating the immune response.

By performing GO and KEGG enrichment analysis on the 326 genes, several immune-relevant signaling pathways were further confirmed to be participated in the response to DTMUV infection, including Cytosolic DNA-sensing pathway, Toll-like receptor signaling pathway, RIG-I-like receptor signaling pathway and NOD-like receptor signaling pathway [65], and so on (Figure 3A and 3B). These antiviral pathways have also been shown to be involved in the infection process of other flaviviruses, such as DENV [66], ZIKV [67], West Nile virus (WNV) [68, 69] and Hepatitis C virus (HCV) [42]. In addition, we found that DTMUV can effectively induce the activation of various immune molecules (*IFN-α2*, *IL12B*, *IRF3*, *DHX58*, *IL-7* and *STAT1*) (Figure 4). Nevertheless, these cytokines did not inhibit DTMUV replication in DEFs (Figure 1C, 1D). This may cause the occurrence of “cytokine storm”, which is the excessive production of multiple inflammatory cytokines to resist pathogens invasion. And overexpressed cytokines will cause great damage to the host cells [70, 71], and finally induce cell death.

The results further showed that genes related to apoptosis, cell cycle arrest, DNA replication and DNA repair were transcriptionally modulated and highly enriched. First, it was found that DTMUV can induce apoptosis of DEFs, and the proportion of apoptosis gradually increases with time (Figure 5A and 5B). Since the percentage of apoptotic cells in DEFs after DTMUV infection was positively correlated with the viral titers achieved by these cells, DTMUV may promote the replication and spread of the virions by inducing cell apoptosis as reported by Dengue virus-2 [72] and PRRSV [73]. In Figure 5C, the dynamic gene expression patterns revealed that the most pro-apoptosis and anti-apoptosis genes showed an early and persistent response. Among the upregulated pro-apoptosis genes, the caspase-7 plays a role in executing apoptosis, moreover, genes closely related to the intrinsic and extrinsic apoptotic pathways were also affected. For instance, caspase-9, cyt-c and apaf-1, which are involved in the formation of apoptosome, were significantly upregulated at all the time points. Meanwhile, death receptor genes (TNFRSF9 [74] and FAS [75]) and caspase-8 were also activated. In addition, Bcl-2 family pro-apoptotic genes (BAK1 [76] and BID [77], inhibitors of IAP family members) participated in the intrinsic apoptosis pathway were signally upregulated as well. The results indicated that both intrinsic and extrinsic apoptosis pathways were initiated after DTMUV infection. What’s more, the pro-apoptotic gene p73 (encoded by TP73), which functions similar to p53, has been reported to be involved in the apoptotic response caused by DNA damage [78], we found it is also involved in DTMUV-induced apoptosis. Moreover, among the downregulated anti-apoptosis genes, NOTCH1 can initiate gene expression programs through translocation of intracellular NOTCH domain (NICD) [79, 80]. In addition, BNIP3 is a member of the Bcl-2 family, it has been found to be located in the nucleus and prevent cell death by inhibiting the expression of AIF (apoptosis inducing factor) [81, 82]. The two downregulated molecules may promote apoptosis caused by DTMUV, thereby increasing the proportion of apoptosis. According to previous reports, apoptosis caused by viral infection is often affected by the innate immune responses, which is considered to be a pioneer in combating RNA virus infection [68, 83–85]. This may explain the activation of the immune-relevant pathways and the high expression of innate immune cytokines observed in this study (Figures 3 and 4), indicating that the intact and functional innate immune responses are involved in the apoptosis process induced by DTMUV, but experimental verification is still required.

Furthermore, our research demonstrated that DTMUV infection caused S phase arrest (Figure 7A and 7B) and repressed vast genes involved in DNA replication (Supplementary Table 1). The S phase arrest may be closely related to the downregulation of some key S phase genes (Figure 7C). Since these genes have a protective effect on DNA damage-induced cytotoxicity, the abrogation of them may promote cell death. Our further found that G2/M arrest occurred at 36 hpi, resulting in insufficient time to repair DNA (Supplementary Table 2). Overall, this reflected that cell cycle arrest and decreased DNA replication/repair capabilities may promote DTMUV invasion of DEFs and cause cell apoptosis.

In addition, many other signaling pathways were also involved in the occurrence of apoptosis [86]. The PPI network revealed that IGF-1 was one of the central nodes (Figure 8). Previous studies have confirmed that IGF-1 can inhibit apoptosis of chicken bursal cells [87], it suggested that IGF-1 may also play an important role in the apoptosis of DEFs. Next, it has been reported that the upregulated anti-apoptosis gene PTGS2 promotes cell survival by activating the PKA and PI3K pathways [88]. Moreover, apoptosis-related genes IL-6 and CFLAR, they are up-regulated at various time points (Figure 5C), indicating that they also play a vital role during apoptosis. In addition to apoptosis-related genes, genes involved in other pathways also play a role in the process of apoptosis, such as STAT1 and TNFAIP3. STAT1 is one of the most critical members of the STAT protein family and plays an important role in regulating cell growth, proliferation and differentiation [89]. Previous studies have confirmed that STAT1 is involved in dsRNA induced apoptosis [90], TNFAIP3 was found to be participate in apoptosis as well [91]. What’s more, STAT1 and TNFAIP3 were both enriched in Toll-like receptor and NOD-like receptor signaling pathway, since both pathways are involved in innate antiviral response, implied that innate immune responses play an essential role in DEFs apoptosis, which are consist with the previous research findings in this article (Figures 3 and 4).

In conclusion, we identified for the first time a dynamic gene expression network for apoptosis and cell cycle arrest induced by DTMUV. Moreover, the apoptotic response was affected by the dynamic expression changes of pro-apoptotic and anti-apoptotic genes, as well as by the intricate interactions with the innate immune cytokines, which provides essential references for deepening our understanding of the responses to DTMUV infection. The PPI network diagram further revealed several pivotal genes involved in apoptosis process (IL-6, STAT1, TNFAIP3, CFLAR, IRF1 and PTGS2), which provides effective information for screening the candidate targets for inhibition of DTMUV.

## MATERIALS AND METHODS

### Cells and viruses

DEFs were obtained from 10-day-old duck embryos according to the manufacturer’s instructions [36]. The cells were grown in Dulbecco’s Modified Eagle’s Medium (DMEM) (Gibco Life Technologies, Shanghai, China) supplemented with 10% newborn bovine serum (NBS) (Gibco, Gaithersburg, MD, USA) at 37°C in a humidified atmosphere with 5% CO_2_. When DEFs reached ~90% confluence, they were mock-infected or infected with DTMUV CQW1 strain (GenBank accession No. KM233707.1) at a multiplicity of infection (MOI) of 1 [37, 38]. After the virus was adsorbed in a 37°C, 5% CO_2_ incubator for 1 h, the inoculum was replaced with maintenance medium (DMEM containing 2% NBS), and the cell samples were collected at 12, 24, 36, 48 and 60 hpi, respectively. Each treatment at each time point has three independent biological repeats.

### Cell viability assays

The cell viability was measured using the CCK8 assay according to the manufacturer’s instructions. Briefly, the cells were seeded in 96-well plates, the cell viability was detected at 12, 24, 36, 48 and 60 hpi, respectively.

### RNA extracting, cDNA library construction and sequencing

Techniques and methods for transcriptome sequencing are provided by Oebiotech (Shanghai, China). Briefly, DEFs treated with PBS or DTMUV for 12, 24, 36, 48 and 60 hpi were collected in biological duplicates and rapidly stored at -80°C until further use. Total RNA was extracted using Trizol reagent (Invitrogen, CA, USA) according to the manufacturer’s instructions. RNA integrity was evaluated using the Agilent Bioanalyzer 2100 (Agilent Technologies, CA, USA), the mRNA was purified using oligo (dT) magnetic beads and then fragmented with fragmentation buffer. The fragmented mRNA was used as a template to synthesize cDNA, and the cDNA libraries was constructed after terminal repair and adding poly (A) and sequencing joints. Then, the libraries were sequenced on the Illumina sequencing platform (HiSeq 2500) to generate 150 bp paired-end reads.

### Data analysis and differentially expressed genes (DEGs)

Clean reads obtained after Raw reads were processed by removing the reads containing adaptors, ploy-N sequences and rRNA. The assembled unigenes were then mapped to mallard (*Anas platyrhynchos*) genome (GenBank: NM_001005484.1) using TopHat2 software [39]. After that, the transcripts were assembled with cufflinks [40]. Differences in genes expression levels were standardized by the reads per kilobase of unigene per million mapped reads (FPKM) method. Genes with a *P* value < 0.05 and |log2 Fold Change|>1 were used to decide the significant levels of DEGs.

**Annotation and function prediction of DEGs**

All DEGs were annotated with GO and KEGG analysis. The GO terms with *P* value less than 0.05 were considered to be remarkable enriched. DEGs identified its main biological functions through GO function enrichment analysis. KEGG is the main database resource of understanding biological systems that links genomes to life (http://www.genome.jp/kegg/tool/map_pathway2.html). Pathways with *P* value less than 0.05 in KEGG were used to recognize the vital metabolic pathways or signal transduction pathways regulated by DEGs.

### Quantitative real-time PCR (Q-RT-PCR)

Isolation of total RNA from DTMUV-infected and mock-infected cells at different time points using Trizol reagent. Purity of all RNA samples were detected by analyzing the A260/A280 ration using a Nano drop ND-1000 spectrophotometer (Nano drop Technologies), which was expected to be 1.8~2.0. First-strand cDNA was obtained from extracted RNA reverse transcribed by PrimeScript^TM^ RT Reagent kit (TAKARA). Q-RT-PCR was performed using SYBR Green real-time PCR assay (CFX96 Bio-Rad, Hercules, CA, USA). The Q-RT-PCR reaction was set up in a total volume of 20 μl containing 2 μl of cDNA, 10 μl of SYBR Premix (Tli RNaseH Plus), 1 μl of forward/reverse primer and 6 μl of ddH_2_O. The duck β-actin gene were used as internal control gene to normalize the targeted gene expression value. DTMUV copies were detected by absolute quantitative PCR according to the real-time quantitative PCR procedure previously established in our laboratory [41]. The quantity mRNA was calculated by the 2^−ΔΔCt^ method and represented as the mean ± SD (n =3). Primers employed are listed in Table 2.

**Table 2:** Primers for Q-RT-PCR analysis of gene expression.

**Target Gene**	**Forward (5'-3')**	**Reverse (5'-3')**
**CCL19**	TTCTCTGCCTCGGTCTC	TTCTCGCTCGTCCTCAG
**IFIH1**	GGTGTCCGCTTATCAGATT	TTGTTGTAGACGCCTTCC
**TRIM25**	CAGCAGTTCTTGGTGTATTG	TTGGTAGCCTTCACATTGG
**CD36**	AAGAGGACCTTACACATACAG	GAGCAGCATTAGGCAACA
**IGF1**	CTTCAGTTCGTATGTGGAGA	TTGTGGTGTAAGCGTCTAC
**SFRP4**	TTCAATGCCGATTCCTCTG	CAACTAGACATCCATCAAGAAG
**Caspase-3**	TGGTGTTGAGGCAGACAGTGGA	CATTCCGCCAGGAGTAATAGCC
**Caspase-7**	AGGCTCCTGGTTTGTGC	AGCGTGGATCATCAGATTG
**Caspase-8**	GGTGATGCTCGTCAGAAAGGTG	AGCCATGCCCAAGAGGAAGT
**Caspase-9**	GCTGCTTCAACTTCCTCCGTAA	CATCTCCACGGACAGACAAAGG
**IFN-α2**	ATCCTCCAACACATCTTCTACA	TCTTAGTTACACATGCCTCCAA
**IL-12B**	CTGAAGAGCACCAGCCAATT	CGTCCAGGTCACTGTTCCA
**IRF3**	AGTGCCTGCTGACCTACCA	TACTGCCGCTGCTTGCTAT
**DHX58**	AAGCCAAGATCAGCGAGAGG	CCAGCGAGACCGTGTAGTAG
**IL-7**	GCCACTACTCCTTGTTCTGTCA	AGCACCTGTCACGATACTCTG
**STAT1**	CCTGTGTCTCTGGAATGATGG	GCTGCTCTCACTGAACCTTAG
**βactin**	GATCACAGCCCTGGCACC	CGGATTCATCATACTCCTGCTT

### Analysis of cell cycle progression

The cells infected with DTMUV for 24 and 36 hpi were collected, washed three times with PBS, then fixed in tubes with 1 ml 70% ice ethanol and incubated overnight at -20°C. After incubation, cells were centrifuged at 1000 r/m. for 5 min, the cell pellets were resuspended in 500 μl of PI/RNASE buffer and incubated for 15 min. The cells were given assay by flow cytometry (FCM) within 1 h.

### Flow cytometric analysis of apoptosis

Based on the instruction of FITC-Annexin V Apoptosis Detection Kit (BD Pharmingen), 5 ul of FITC-Annexin V and 5 ul PI were added to 100 ul of cell suspension, and then incubate for 15 min at 25 °C in the dark. 400 ul of 1×Binding Buffer was added to each tube and the percentage of apoptotic cells were assayed by FCM within 1 h.

### Caspases activity assays

The activities of caspase-3/7, caspase-9 and caspase-8 were measured using Caspase-Glo assay kit (Promega, CA, USA). Briefly, approximately 20,000 cells (with or without DTMUV infection) were collected at 12, 24, 36, 48 and 60 hpi, then added to a 96-well plate containing 100 ul of Caspase-Glo reagent and incubated for 30 min. Finally, the luciferase activity was detected at 485/530 nm using a multifunctional enzyme marking instrument (Thermo Scientific, USA), and the fold change in protease activity was measured via comparing the luciferase activity of infected cells with that of mock-infected cells.

### Statistical analysis

All experiments were performed in triplicate and the data analysis was performed using GraphPad Prism 7.0. The results are expressed as the mean ± SEM and statistical significance was assessed with Student’s *t*-test. *P* values less than 0.05 were considered to be statistically significant.

## Supplementary Material

Supplementary Figures

Supplementary Tables

Supplementary Dataset 1

Supplementary Dataset 2

Supplementary Dataset 3

Supplementary Dataset 4
