# Supplementary material for: Transcriptome analysis of duck embryo fibroblasts for the dynamic response to duck tembusu virus infection and dual regulation of apoptosis genes
Source: Aging (Albany NY). 2020 Sep 7;12(17):17503–27. doi: 10.18632/aging.103759 (PMC7521532; doi:10.18632/aging.103759)
Supplement: Supplementary Figures [file aging-12-103759-s004..pdf]

SUPPLEMENTARY FIGURES

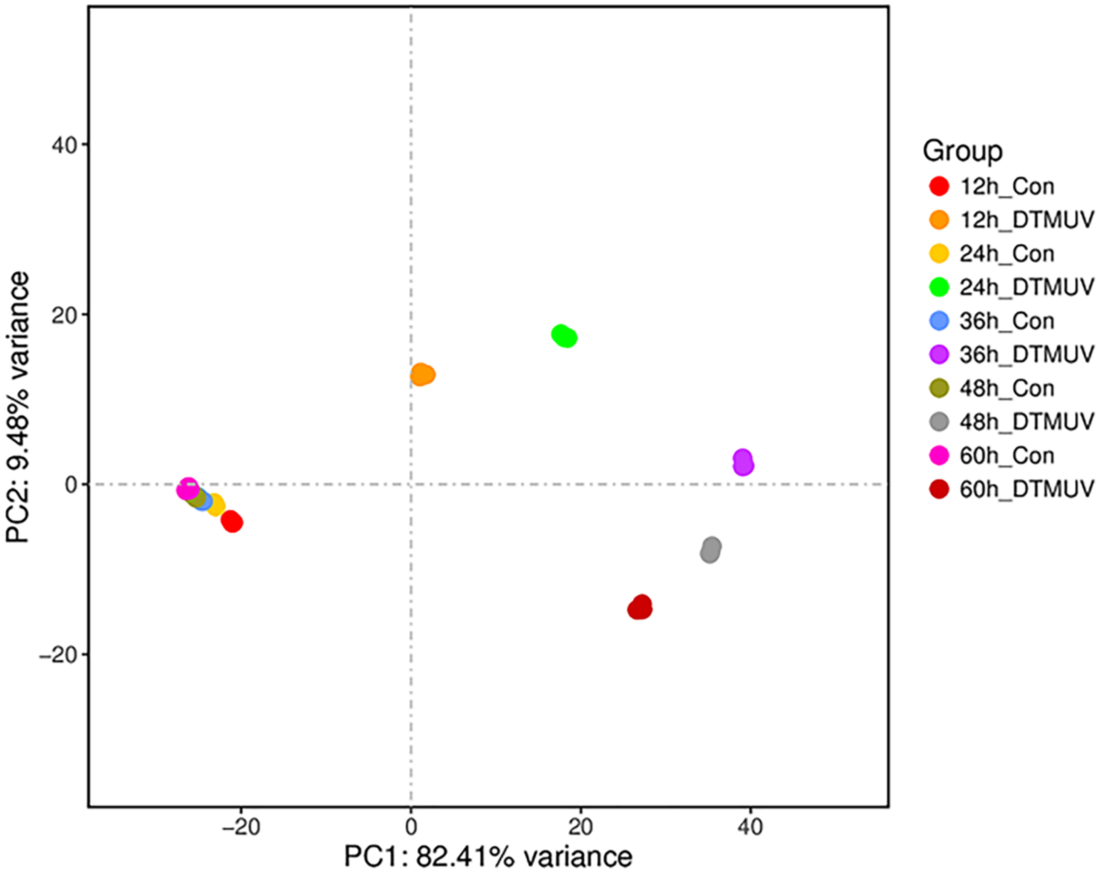

Supplementary Figure 1. Principal component of samples at 12, 24, 36, 48 and 60 hpi. Note: PC1 shows the differences among duck Tembusu virus (DTMUV)-infected samples; PC2 indicates differences between mock- and DTMUV-infected samples. 12h_Con, mock-infected DEFs at 12 hpi; 24h_Con, mock-infected DEFs at 24 hpi; 36h_Con, mock-infected DEFs at 36 hpi; 48h_Con, mock-infected DEFs at 48 hpi; 60h_Con, mock-infected DEFs at 60 hpi; 12h_DTMUV, DTMUV-infected DEFs at 12 hpi, 24h_DTMUV, DTMUV-infected DEFs at 24 hpi, 36h_DTMUV, DTMUV-infected DEFs at 36 hpi, 48h_DTMUV, DTMUV-infected DEFs at 48 hpi, 60h_DTMUV, DTMUV-infected DEFs at 60 hpi.

A

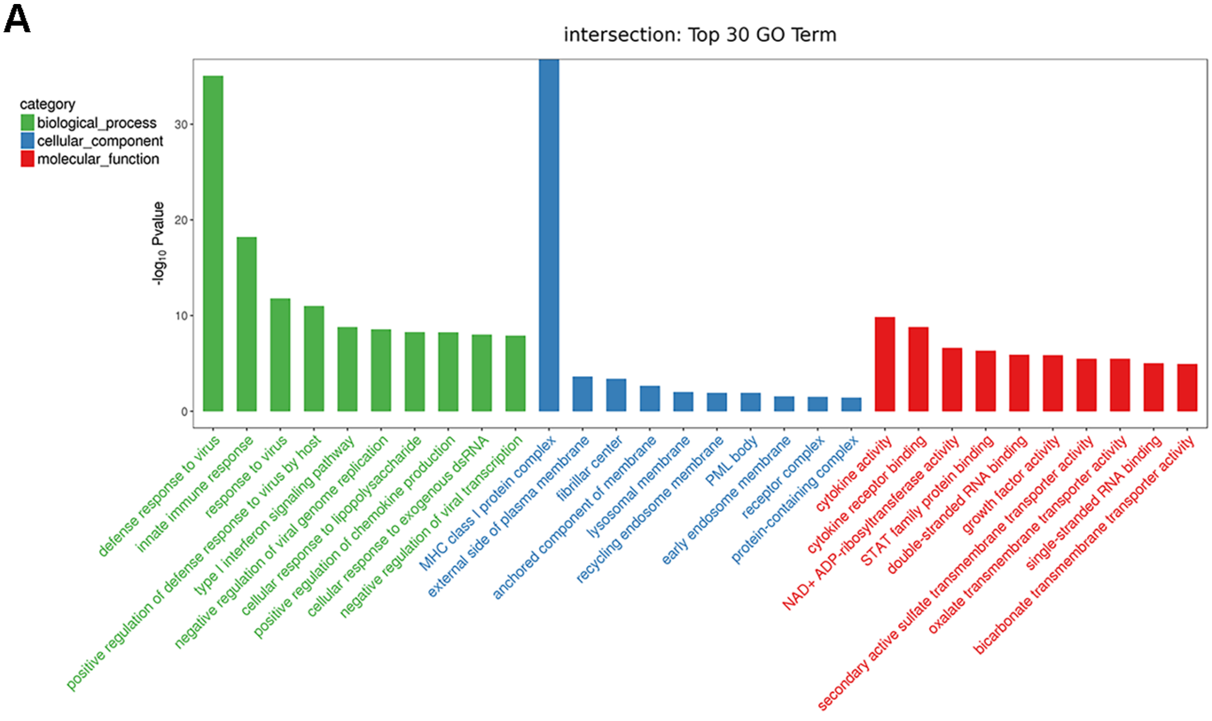

B

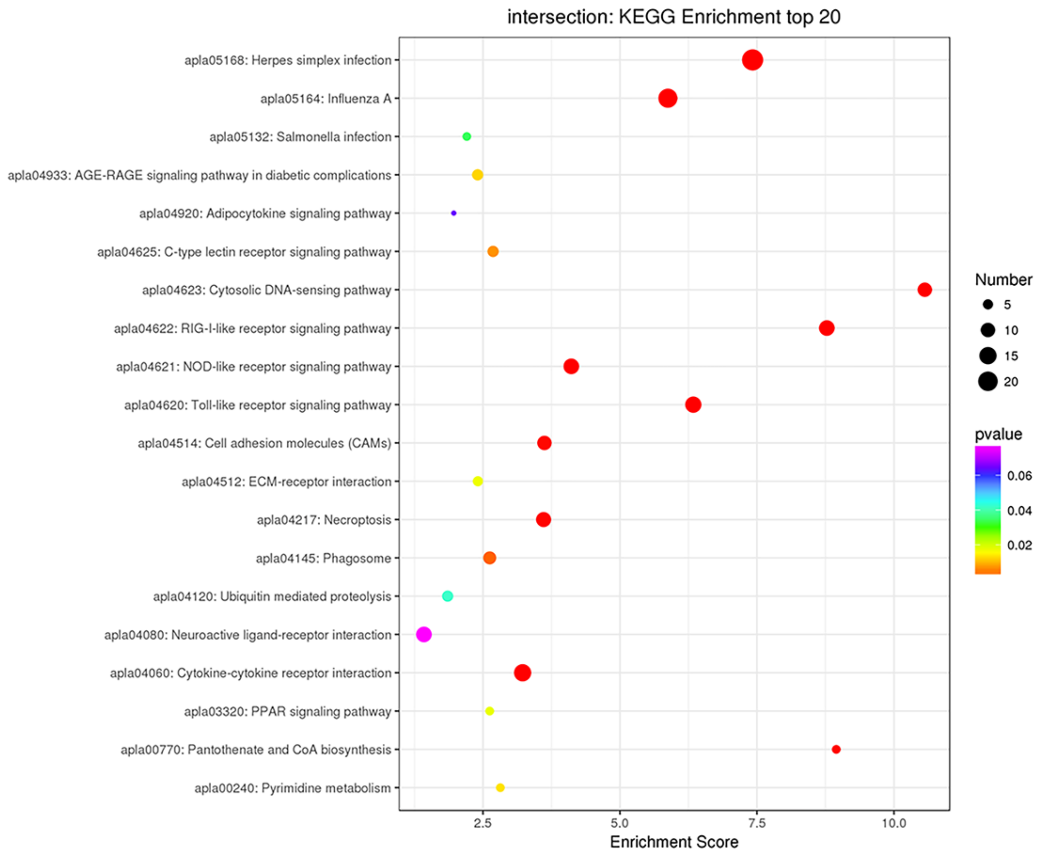

Supplementary Figure 2. Analysis of the upregulated genes with expression changes at all time points. (**A**) The top 30 Gene Ontology (GO) enrichment of differentially expressed genes. (**B**) The top 20 Kyoto Encyclopedia of Genes and Genomes (KEGG) enrichment analysis of differentially expressed genes.

A

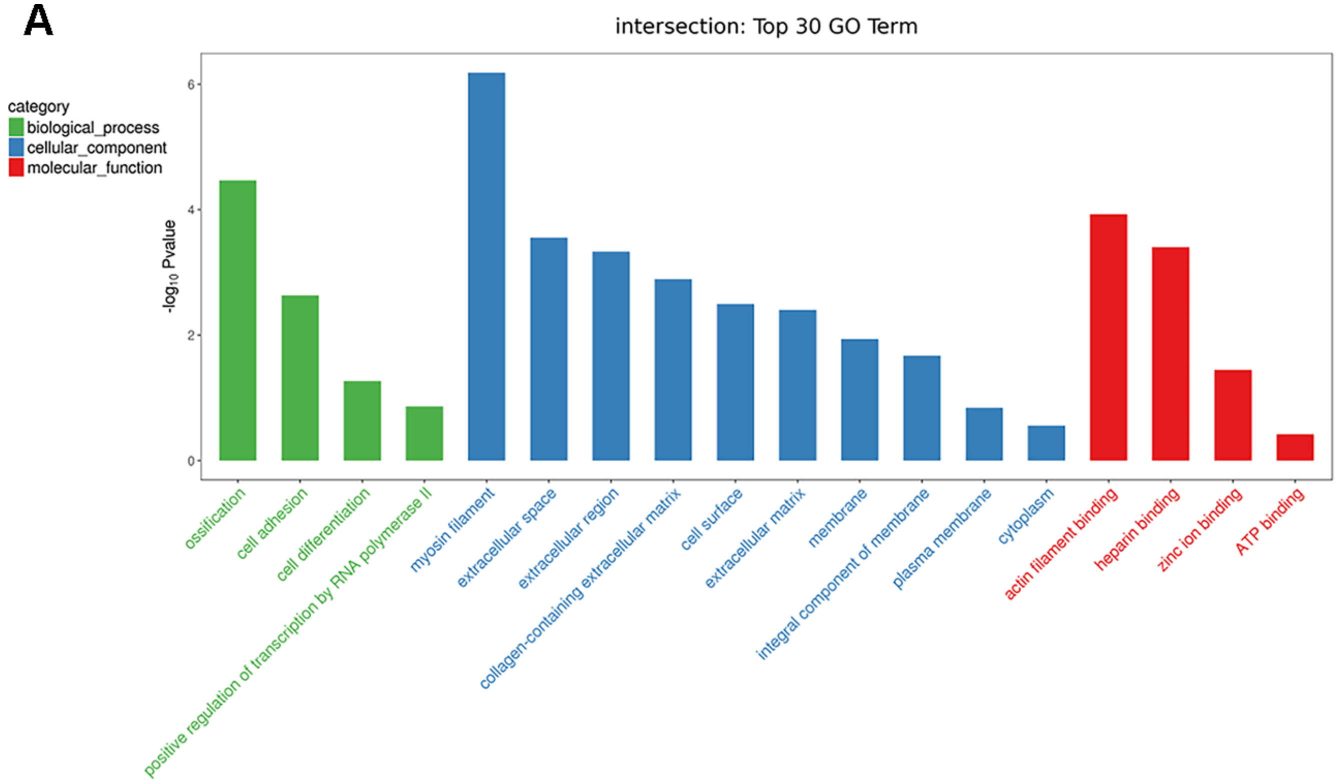

B

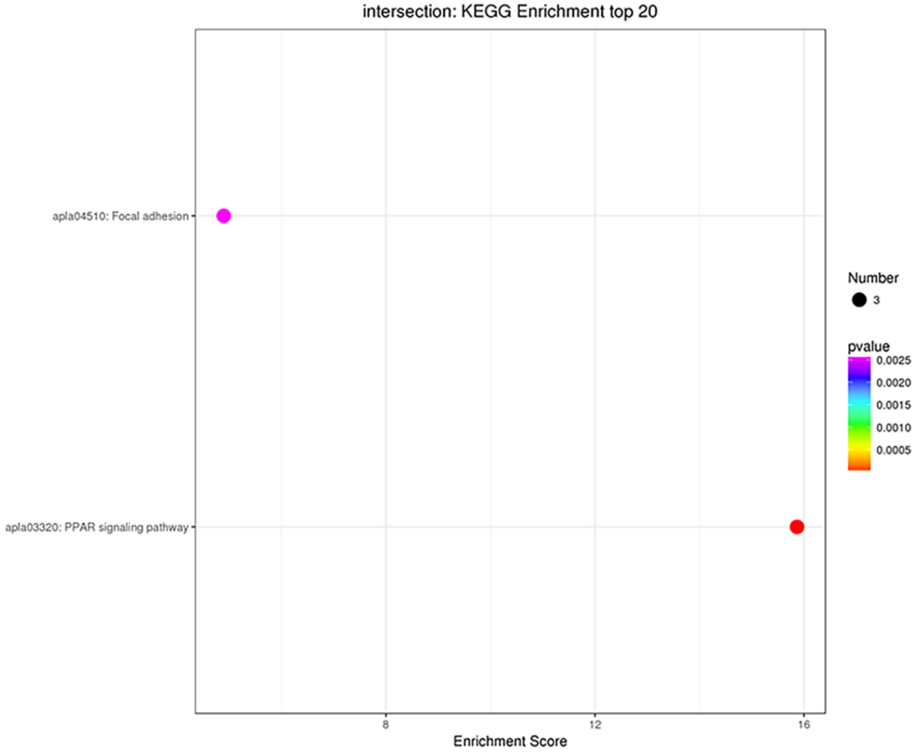

Supplementary Figure 3. Analysis of the downregulated genes with expression changes at all time points. (**A**) The top 30 Gene Ontology (GO) enrichment of differentially expressed genes. (**B**) The top 20 Kyoto Encyclopedia of Genes and Genomes (KEGG) enrichment analysis of differentially expressed genes.
