# Supplementary material for: Transcriptome analysis of duck embryo fibroblasts for the dynamic response to duck tembusu virus infection and dual regulation of apoptosis genes
Source: Aging (Albany NY). 2020 Sep 7;12(17):17503–27. doi: 10.18632/aging.103759 (PMC7521532; doi:10.18632/aging.103759)
Supplement: Supplementary Tables [file aging-12-103759-s003..pdf]

SUPPLEMENTARY TABLES

Supplementary Table 1. Information of DNA replication related genes.

Gene Name	Putative function (cell component, function and process)	12hpi	24hpi	36hpi	48hpi	60hpi
		Log$_2$(fold change)				
BLM	ATP-dependent DNA helicase activity; DNA replication initiation	-0.43	-0.38	-0.94	-1.53	0.43
BRCA2	structure-specific DNA binding; transcription, DNA-dependent	-0.15	-0.62	-1.11	-1.06	0.16
CDT1	S phase of mitotic cell cycle; DNA-dependent DNA replication initiation	-0.09	0.35	-0.22	-0.06	0.11
DKC1	RNA-directed DNA polymerase activity; rRNA processing;	0.10	-0.49	-0.34	-0.06	0.17
DNA2	DNA helicase activity; DNA stand elongation	-0.08	0.22	0.12	-0.63	0.43
DSCC1	DNA replication; mitotic sister chromatid cohesion	-0.12	-0.54	-0.68	-0.47	0.20
GINS2	the initiation of DNA replication; DNA-dependent DNA replication	-0.06	-0.14	-0.54	-0.55	1.01
HMGB1	negative regulation of transcription from RNA polymerase II promoter; DNA conformation change	0.24	-0.17	-0.47	-0.59	0.21
MCM4	helicase activity; DNA-dependent DNA replication;	-0.02	-0.02	-0.59	-0.53	0.15
MCM5	Regulation of gene expression	-0.19	0.26	0.21	-0.38	0.16
POLA1	DNA-directed RNA polymerase activity; structure-specific DNA binding; DNA strand elongation involved in DNA replication	0.02	-0.08	-0.24	-0.48	0.07
POLD3	DNA replication; DNA repair	0.11	0.04	-0.24	-0.29	0.19
PRIM1	DNA polymerase, primase complex; replication fork	0.05	0.14	-0.71	-0.59	0.93
RAD51	DNA-dependent ATPase activity; DNA-dependent DNA replication	-0.05	0.03	-0.51	-2.05	1.47
RFC2	DNA metabolic process; DNA clamp loader activity	0.01	0.03	-0.63	-1.06	0.94
RFC3	nucleoside-triphosphatase activity; DNA-dependent DNA replication; DNA repair	-0.04	-0.08	-0.63	-0.91	0.48
RFC5	DNA metabolic process; DNA clamp loader activity	-0.34	-0.06	-0.07	0.25	0.01
RPA1	structure-specific DNA binding; DNA replication; nucleotide-excision repair	0.24	0.65	-0.22	-0.53	0.23
SMC3	motor activity; DNA replication; chromosome segregation;	0.34	0.13	-0.05	-0.33	0.40
TERT	sequence-specific DNA binding; RNA-dependent DNA replication	-0.30	-0.07	-1.37	-0.19	1.42
TFAM	DNA-dependent DNA replication; mitochondrial transcription factor	0.00	-0.07	-0.06	-0.63	0.87
TIPIN	DNA replication involved in S phase;	0.12	0.17	-0.09	-0.21	0.28

Supplementary Table 2. Information of DNA repair related genes.

Gene Name	Putative function (cell component, function and process)	12hpi	24hpi	36hpi	48hpi	60hpi
		Log$_2$(fold change)				
XPC	DNA secondary structure binding; nucleotide-excision repair	0.20	0.04	-0.63	-0.82	-0.47
ERCC6	helicase activity; nucleotide-excision repair; RNA elongation	-0.08	-0.34	-0.78	-0.30	-0.47
DDB2	nucleotide-excision repair	-0.12	-0.14	0.19	0.27	0.19
RPA1	recombinational repair; DNA replication; nucleotide-excision repair	0.24	0.65	-0.22	-0.53	-0.23
RPA2	structure-specific DNA binding; DNA replication; nucleotide-excision repair	0.03	-0.08	-0.04	0.03	0.22
RPA3	structure-specific DNA binding; nucleotide-excision repair	0.41	0.15	0.53	0.13	0.44
XRCC2	ATPase activity, coupled; recombinational repair	-0.23	-0.07	-0.36	-0.58	-0.45
RAD51	DNA-dependent ATPase activity; recombinational repair	-0.05	0.03	-0.51	-2.05	-1.47
RAD54L	nucleoside-triphosphatase activity; recombinational repair	-0.23	0.27	-0.70	-0.29	-0.22
RAD54B	helicase activity; recombinational repair	-0.01	0.03	-0.75	-0.41	-0.33
RAD51AP1	structure-specific DNA binding; recombinational repair	0.27	0.86	0.03	0.57	0.39
BRCA2	structure-specific DNA binding; recombinational repair; transcription, DNA-dependent	-0.15	-0.62	-1.11	-1.06	0.16
BRCA1	recombinational repair; regulation of gene-specific transcription from RNA polymerase II promoter	-0.25	-0.33	-0.70	-1.09	-0.43
EME1	Homologous recombination	-0.21	0.11	-0.24	-0.39	-0.66
XRCC4	DNA ligation; non-homologous recombination	0.31	0.38	-0.24	-0.31	-0.06
PRKDC	Non-homologous recombination	-0.04	-0.16	-0.46	-0.07	-0.03
MSH2	single base insertion or deletion binding; sequence-specific DNA binding; mismatch repair	0.11	-0.20	0.06	0.48	1.10
MSH6	regulation of DNA recombination; mismatch repair	0.28	0.22	-0.18	-0.55	-0.10
EXO1	single-stranded DNA specific exodeoxyribonuclease activity;mismatch repair	0.02	-0.10	0.67	-0.55	0.20
MUTYH	nuclease activity; base-excision repair, AP site formation	-0.79	-0.09	-0.89	-0.83	0.48
FEN1	endodeoxyribonuclease activity; structure-specific DNA binding; base excion repair	-0.16	0.13	-0.15	0.02	0.36
NEIL3	DNA secondary structure binding; base excision repair	-0.17	-0.20	0.39	0.07	-1.12
OGG1	oxidized base lesion DNA N-glycosylase activity; base-excision repair, AP site formation	-0.12	0.22	0.57	-0.27	-0.15
HMGB1	base-excision repair; regulation of gene-specific transcription from RNA polymerase II promoter	0.24	-0.17	-0.47	-0.59	-0.21
HMGB2	base-excision repair; regulation of gene-specific transcription from RNA polymerase II promoter	0.53	0.99	1.24	0.59	0.97
PMS1	nucleoside-triphosphatase activity; DNA repair; DNA recombination	0.38	0.01	0.09	-0.22	-0.09
FANCC	DNA repair	-0.15	0.34	0.96	0.06	0.98
USP1	endopeptidase activity; DNA repair;	0.10	0.01	-0.06	-0.06	0.29
RBBP8	endodeoxyribonuclease activity; DNA double-strand break processing	0.25	0.12	-0.26	-0.59	0.34
DCLRE1C	exonuclease activity; DNA repair	0.16	-0.10	-0.52	-0.98	-0.57
RFC3	nucleoside-triphosphatase activity; DNA-dependent DNA replication; DNA repair	-0.04	-0.08	-0.63	-0.91	-0.48
MAD2L2	nucleotidyltransferase activity; translesion synthesis	-0.17	-0.04	-0.26	-0.41	-0.45
BLM	ATP-dependent DNA helicase activity; DNA damage checkpoint; recombinational repair;	-0.43	-0.38	-0.94	-1.53	-0.43
POLA1	DNA-directed RNA polymerase activity; DNA strand elongation involved in DNA replication	0.02	-0.08	-0.24	-0.48	-0.07
TRIP13	adenyl ribonucleotide binding; DNA repair; DNA recombination;	0.21	0.04	-0.36	-1.07	-0.48
BRIP1	DNA helicase activity; DNA repair; regulation of transcription, DNA-dependent	0.13	-0.14	-0.45	-0.58	-0.10
TP73	sequence-specific DNA binding; DNA repair; regulation of gene-specific transcription from RNA polymerase II promotertransduction by p53 class mediator	-0.38	1.12	1.78	0.85	0.03
TUBB6	nucleoside-triphosphatase activity; DNA repair	0.00	0.25	-0.64	-1.38	-1.78
POLD3	DNA replication; DNA repair	0.11	0.04	-0.24	-0.29	-0.19
TDP1	nuclease activity; structure-specific DNA binding; DNA repair	0.08	0.21	0.78	1.06	1.79
PARP1	nucleotide binding; nucleic acid binding; DNA repair; transcription, DNA-dependent	-0.01	-0.15	-0.93	-1.00	-1.01
